# Mit dem Rettungsdienst direkt in die Arztpraxis – eine wirkungsvolle Entlastung der Notaufnahmen? Analyse von notfallmedizinischen Routinedaten aus Präklinik und Klinik

**DOI:** 10.1007/s00063-021-00860-x

**Published:** 2021-09-01

**Authors:** Tobias Lindner, Alessandro Campione, Martin Möckel, Cornelia Henschke, Janosch Dahmen, Anna Slagman

**Affiliations:** 1 Notfall- und Akutmedizin, Charité – Universitätsmedizin Berlin Campus Virchow-Klinikum und Campus Mitte, Augustenburger Platz 1, 13353 Berlin, Deutschland; 2 Fachgebiet Management im Gesundheitswesen, Technische Universität Berlin, Berlin, Deutschland; 3 Fakultät für Gesundheit, Department Humanmedizin, Universität Witten/Herdecke, Witten, Deutschland; 4 Ärztliche Leitung Rettungsdienst, Berliner Feuerwehr, Berlin, Deutschland

**Keywords:** Notfallversorgung, Ambulante Behandlung, Dringlichkeitseinschätzung, Krankenhauszuweisung, Emergency care, Outpatient, Triage, Hospital admission

## Abstract

**Hintergrund:**

Das Sachverständigengutachten zur bedarfsgerechten Steuerung der Gesundheitsversorgung 2018 empfiehlt zur Entlastung der klinischen Notfallversorgung unter anderem, dem Rettungsdienst die Option einzuräumen, geeignete Patienten direkt in eine Praxis zur fachärztlichen Versorgung zu transportieren.

**Fragstellung:**

Quantifizierung von Patienten, die mit dem Rettungswagen (RTW) in der Notaufnahme vorgestellt wurden und sicher und sinnvoll zur Behandlung primär in eine Praxis transportiert hätten werden können.

**Material und Methoden:**

Retrospektive Auswertung prähospitaler und klinischer Daten von erwachsenen Patienten, die innerhalb von 2 Monaten mit einem RTW in die Notaufnahme eines universitären Maximalversorgers eingeliefert wurden. Anhand einer durch Rettungsassistenten durchgeführten, 5‑stufigen Dringlichkeitseinschätzung erfolgte durch die Autoren zunächst die Kategorisierung in „dringliche“ (Arztkontakt innerhalb von maximal 30 min notwendig) und „weniger dringliche“ Fälle (Arztkontakt nicht in weniger als 30 min notwendig, maximal in 120 min). In der Gruppe der „weniger dringlichen“ Fälle wurden aus den klinischen Behandlungsdaten diejenigen mit ambulanter Weiterbehandlung diskriminiert sowie folgend die Fälle, deren administrative Notaufnahme von Montag bis Freitag (Feiertage ausgeschlossen) jeweils zwischen 08.00 und 19.00 Uhr stattfand (praxistaugliche Fälle). Außerdem erfolgte eine medizinisch-inhaltliche Differenzierung dieser Fälle und ein Vergleich mit der Dringlichkeitseinschätzung in der Notaufnahme (Manchester Triage System, MTS).

**Ergebnisse:**

Es wurden *n* = 1260 Patienten mit dem RTW in die Notaufnahme disponiert (Gesamtbehandlungszahl *n* = 11.506). Bei *n* = 894 war eine prähospitale Dringlichkeitseinschätzung dokumentiert, auf deren Grundlage *n* = 477 (53,4 %) als „weniger dringliche“ Fälle kategorisiert und *n* = 317 (66,5 %) ambulant weiterbehandelt wurden, *n* = 114 (23,9 %) zu üblichen Praxisöffnungszeiten. Das entspricht 1 % aller im Beobachtungszeitraum behandelten Patienten. 70 Fälle dieser praxistauglichen Gruppe (63,6 % von *n* = 110 mit dokumentierter MTS) wurden in der Notaufnahme dringlicher eingestuft. Die prähospital dokumentierten Beschwerdebilder und die in der Klinik erhobenen Hauptdiagnosen lassen den Einsatz relevanter diagnostischer Ressourcen bei einer Vielzahl der praxistauglichen Fälle vermuten.

**Diskussion:**

Die Notaufnahmen könnten im Zeitfenster üblicher Praxisöffnungszeiten bei primärer Disposition der weniger dringlichen, ambulant behandelten Fälle in eine Praxis von ungefähr jedem zehnten mit dem RTW disponierten Patienten und 1 % ihrer Gesamtpatientenzahl entlastet werden. Unter dem Aspekt der Patientensicherheit ist dieses Vorgehen mit > 60 % möglicher Untertriage kritisch zu bewerten. Für die Diagnostik und Behandlung müssten entsprechende Ressourcen in der Praxis vorhanden und dem Rettungsdienst bekannt sein. Die primäre Disposition in eine Praxis erscheint bezogen auf die mögliche Entlastung einer großstädtischen Notaufnahme unbedeutend, ist potenziell patientengefährdend und mit einem enormen logistischen Aufwand verbunden.

## Abstract (en)

**Background:**

In the 2018 advisory opinion concerning the realignment of healthcare, it is advocated that in order to relieve pressure on emergency departments (ED) prehospital medical emergency services should be given the option to directly transport suitable patients to doctors’ offices.

**Objectives:**

To determine the prevalence of patients treated by prehospital emergency services that have the potential to be directly allocated to a primary care provider.

**Materials and methods:**

Preclinical and clinical data of adult patients who in a 2-month period were transported to the ED of a university hospital by an ambulance were evaluated. To determine a safe and meaningful transport directly to a doctor’s office, a stepwise assessment was carried out: patients were categorized on the basis of the prehospital assessment of urgency as “urgent” (contact to doctor necessary within a maximum time of 30 min) and “less urgent” (contact to doctor not necessary within 30 min, maximum 120 min). “Less urgent” patients were further divided and those treated as outpatients were identified. This group was further restricted to cases whose administrative reception in the ED was documented Monday–Friday between 8 am and 7 pm. In addition, these cases were further differentiated with regard to medical content and compared with the triage results in the ED (Manchester Triage, MTS).

**Results:**

In all, 1260 patients were brought to the ED by ambulance within the study period (total number of patients treated in this time period *n* = 11,506); 894 cases had a documented prehospital level of urgency and could therefore be included. Of these *n* = 477 (53.4%) were categorized as “less urgent”; 317 (66.5%) of these “less urgent” cases were treated as outpatients in the ED, and *n* = 114 (23.9%) in a time frame potentially suitable for direct transport to doctors’ offices, which is 1% of all patients treated in the ED in the time period examined. However, 70 of the cases suitable for doctors’ office (63.6% of *n* = 110 with documented MTS) were rated more urgent in the ED. With regards to prehospital complaints and documented diagnosis we assume employment of a relevant amount of resources in the treatment of these cases.

**Conclusions:**

EDs could be relieved from every tenth patient brought in by prehospital emergency services (1% of all patients treated) during normal offices hours by direct allocation to doctors’ offices. Regarding patient’s safety this process however has to be seen critically as > 60% of these cases were potentially undertriaged. Necessary resources for diagnostics and treatment have to be available in the doctors’ offices and known to prehospital emergency services. Primary assignment of patients to doctors’ offices by prehospital emergency can only relieve urban EDs to a negligible extent, is potentially dangerous and linked to a tremendous logistic effort.

## Hintergrund und Fragestellung

In Sachverständigengutachten zur „Bedarfsgerechten Steuerung der Gesundheitsversorgung“ von 2018 [4] heißt es auf S. 548 „[…] Die niedergelassenen Ärzte behandeln Patienten mit dringlichem ambulantem Behandlungsbedarf in ihren Praxen oder über den vertragsärztlichen Bereitschaftsdienst der Kassenärztlichen Vereinigungen (KVen), der die ambulante Versorgung außerhalb der regulären Praxisöffnungszeiten sicherstellen soll. Der Rettungsdienst ist vor allem für lebensrettende Sofortmaßnahmen bzw. für die Verhinderung schwerer gesundheitlicher Schäden sowie für den qualifizierten Transport ins Krankenhaus zuständig. Die Notaufnahmen der Krankenhäuser wiederum versorgen die Patienten aus dem Rettungsdienst weiter. Sie sind aber auch direkte Anlaufstelle für Patienten, wobei hier eine stationäre Behandlungsoption oder deren Ausschluss im Fokus steht […]“. Zur Entlastung der oft überfüllten klinischen Notfallversorgung oder einer zu schaffenden zentralen Anlaufstelle wird weiter empfohlen, dem Rettungsdienst die Option des Patiententransporte direkt in eine Praxis zu gewähren.

Dieses setzt voraus, dass es für den Rettungsdienst möglich ist, die Patienten am Einsatzort zu erkennen, bei denen ein solcher Transport nicht nur sicher, sondern auch sinnvoll ist, da höchstwahrscheinlich eine ambulante Weiterbehandlung erfolgt. Zusätzlich muss eingeschätzt werden, ob die zur Diagnostik und Behandlung notwendigen Ressourcen in ausschließlich ambulanten Versorgungsstrukturen vorhanden sind.

Primäres Ziel dieser Arbeit ist es, auf Basis vorhandener prähospitaler und klinischer Daten zu prüfen, welcher Anteil der durch von einem Rettungstransportwagen (RTW) zugewiesenen Patienten sich nach der im Sachverständigengutachten ausgesprochenen Empfehlung potenziell eignen würde, direkt in eine rein ambulante Versorgungsstruktur transportiert zu werden und ob hierdurch eine sinnvolle Entlastung der Notaufnahmen zu erwarten wäre. Sekundär wird die prähospitale und klinische Dringlichkeitseinschätzung hinsichtlich der Konkordanz überprüft.

## Material und Methodik

### Studiendesign

Über den Zeitraum von 2 Monaten (01.05.2014 bis 30.06.2014) wurde die prähospitale Einsatzdokumentation und die klinische Dokumentation aller erwachsenen Patienten, die mit dem Rettungsdienst in die Notaufnahme des Campus Virchow-Klinikum der Charité – Universitätsmedizin Berlin eingeliefert wurden, erfasst und in eine SPSS-Datenbank (IBM Corp., Armonk, NY, USA, released 2013. IBM SPSS Statistics for Windows, Version 22.0) übertragen. Es wurden nur die Einsatzdokumentationen der Zuweisungen mit dem RTW (Rettungswagen) ausgewertet. Die prähospitalen Daten lagen ausschließlich in Form einer handschriftlichen Dokumentation vor. Die Dokumentation in der Notaufnahme ist vollständig digitalisiert. Die korrespondierenden klinischen Daten (Eintreffdatum, Eintreffzeit, Ersteinschätzung in der Notaufnahme, Behandlungsart *[ambulant vs. stationär]* und Krankenhaushauptdiagnose) wurden automatisch aus dem Krankenhausinformationssystem extrahiert und in der Datenbank zusammengeführt. Die Analysen erfolgten retrospektiv und pseudonymisiert. Es war keine Einwilligung der Patienten möglich und notwendig. Die Ethikkommission der Charité hat der Studie zugestimmt (EA1/172/14).

### Prähospitale Dringlichkeitseinschätzung

Im Jahr 2014 konnte in den Rettungsdienstprotokollen der Berliner Feuerwehr eine Dringlichkeitseinschätzung in den nichtnotärztlichen Einsatzprotokollen (Diagramm Halbach GmbH & Co. KG, Schwerte, Deutschland; 176.101.18.142, Formular ID: 13Y118U4, BF, DIRE, Version2.0) dokumentiert werden. In der Box „Dringlichkeit“ wurden 5 Stufen unterschieden und waren jeweils markierbar: „nicht dringlich (max. 120 min)“, „normal (max. 90 min)“, „dringlich (max. 30 min)“, „sehr dringlich (max. 10 min)“, „Lebensgefahr (Sofortbehandl.)“. Es erfolgte keine explizite Schulung der Rettungsdienstkräfte zur Nutzung der Dringlichkeitseinschätzung durch die Berliner Feuerwehr. Eine Ausfüllhilfe bzw. hinterlegte Zuordnungskriterien sind nicht in dem Protokoll vermerkt.

Auf Basis dieser 5‑stufigen Dringlichkeitseinschätzung wurde die Gesamtpopulation nach Festlegung durch die Autoren in 2 Dringlichkeitskategorien unterteilt. Als Grundlage dieser Aufteilung diente die Überlegung, dass bei weniger dringlichen Fällen ein Arztkontakt nicht unter 30 min notwendig sein sollte, was auch in der Niederlassung eines einzelnen Arztes umsetzbar sein sollte. Die maximale Zeit bis zum Arztkontakt sollte 120 min dabei nicht überschreiten.

#### Gruppe A („dringliche Fälle“)

Die 3 Einschätzungskategorien „Lebensgefahr (Sofortbehandl.)“, „sehr dringlich (max. 10 min)“ und „dringlich (max. 30 min)“ wurden als Fälle zusammengefasst, die auf Basis der Dringlichkeit primär einer Notaufnahme zuzuweisen wären (Tab. 1).

**Table 1:** 

Prähospitale Dringlichkeit	Gruppe	Anteil an allen prähospital eingestuften Patienten (*n* = 894)	Art der Weiterbehandlung	
			Stationär	Ambulant
Lebensgefahr (Sofortbehandlung)	A	2,3 % (*n* = 21)	90,5 % (*n* = 19)	9,5 % (*n* = 2)
Sehr Dringlich (max. 10 min)		8,4 % (*n* = 75)	76,0 % (*n* = 57)	24,0 % (*n* = 18)
Dringlich (max. 30 min)		35,9 % (*n* = 321)	47,4 % (*n* = 152)	52,6 % (*n* = 169)
**Normal (max. 90** **min)**	**B**	**45,8** **% (*n*** **=** **409)**	**35,0** **% (*n*** **=** **143)**	**65,0** **% (*n*** **=** **266)**
**Nicht dringlich (max. 120** **min)**		**7,6** **% (*n*** **=** **68)**	**25,0** **% (*n*** **=** **17)**	**75,0** **% (*n*** **=** **51)**
*Gesamt*	*Alle*	*100* *% (n* *=* *894)*	*43,4* *% (n* *=* *388)*	*56,6* *% (n* *=* *506)*

#### Gruppe B („weniger dringliche Fälle“)

Die beiden am Einsatzort als „normal (max. 90 min)“ und „nicht dringlich (max. 120 min)“ eingeschätzten Fälle wurden als potenziell einer niedergelassenen Arztpraxis zuführbar angesehen und als „weniger dringliche Fälle“ zusammengefasst.

Die Gruppe B wurde dann auf Basis der in der klinischen Dokumentation festgehaltenen Art der Weiterbehandlung in der Notaufnahme in stationäre und ambulante Weiterbehandlungsfälle unterteilt. Die ambulanten Weiterbehandlungsfälle wurden dann weiter untergruppiert in die Fälle, die Montag bis Freitag zwischen 8.00 und 19.00 Uhr (ohne Feiertage) in der Notaufnahme behandelt wurden, und Fälle, die nicht in diese Zeiten fielen. Als Grundlage diente das im Krankenhausinformationssystem dokumentierte Datum und die Zeit der administrativen Aufnahme.

### Medizinisch-inhaltliche Bewertung

Eine medizinisch-inhaltliche Bewertung erfolgte für die Patienten der Gruppe B mit ambulanter Weiterbehandlung, die potenziell in die Praxis gebracht hätten werden können (d. h. Eintreffzeit in der Notaufnahme zu Praxisöffnungszeiten: Montag bis Freitag, jeweils 8.00 bis 19.00 Uhr ohne Feiertage).

#### Prähospital

Zur prähospitalen, medizinisch-inhaltlichen Bewertung wurden die Boxen „Erscheinungsbild“ und „Art der Verletzung“ der Rettungsdienstprotokolle ausgewertet.

Die Box „Erscheinungsbild“ ist in 3 Spalten untergegliedert, die mit „Internistisch“, „Neurologisch“ und „Sonstige“ überschrieben sind. Unter „Internistisch“ sind einzeln markierbar: „akutes Koronarsyndrom“, „Lungenödem“, „Synkope“, „Asthma/COLD“, „BZ-Entgleisung“, „GI-Blutung“ und „sonst. int. Notfall“ (mit Option freier Textangabe).

Unter „Neurologisch“ sind an Erkrankungen „TIA/Apoplex“, „Krampfanfall“ und „Nackensteifigkeit“ aufgeführt gefolgt von neurologischen Untersuchungsbefunden (wurden nicht ausgewertet). Unter „Sonstige“ sind „chir./orthopäd. Erkrankung“, „gyn./Geburt“, „pädiatrisch“, „Vergiftung“ und „sonstiger Notfall“ (mit Option der freier Textangabe).

Im Feld „Art der Verletzungen“ können Angaben zum „Unfallmechanismus“, zum „Verletzungshergang“, zu „Verbrennungen/Verbrühungen“ und zur anatomischen Lokalisation mit jeweiliger Verletzungsschwere gemacht werden. Ausgewertet wurde nur die anatomische Lokalisation, wobei differenzierbar war: „Schädel-Hirn“, „Gesicht“, „HWS“, „Thorax“, „Abdomen“, „BWS/LWS“, „Becken“, „obere Extremit.“, „untere Extremit.“ sowie „Weichteile“ und eine angegebene „Verbrennung/Verbrühung“.

#### Klinisch

Die klinische, medizinisch-inhaltliche Bewertung der „weniger dringlichen“ Fälle erfolgte auf Basis der dokumentierten Hauptdiagnose.

### Klinische Ersteinschätzung

Die prähospitale Dringlichkeitseinschätzung wurde mit der zeitlich im Behandlungsverlauf nachgelagerten, ebenso 5‑stufigen, klinischen Ersteinschätzung in der Notaufnahme (Manchester Triage System, MTS) verglichen. Nach der Zuordnung zu einem von 52 Leitsymptomen erfolgt innerhalb des Leitsymptom eine Dringlichkeitseinschätzung bezogen auf die maximale Zeitdauer bis zum ärztlichen Erstkontakt. Es werden 5 Dringlichkeitsstufen unterschieden: rot = sofort (0 min), orange = sehr dringend (10 min), gelb = dringend (30 min), grün = normal (90 min), blau = nicht dringend (120 min). Für die Dringlichkeitseinschätzung können generelle Parameter (wie z. B. Lebensgefahr, Schmerz, Bewusstseinszustand), aber auch für das Leitsymptom spezifische Indikatoren gewählt werden.

### Statistik

Statistische Berechnungen wurden mittels RStudio Version 1.1.456 für Windows durchgeführt. Verteilungen quantitativer Merkmale wurden vor Durchführung der Tests geplottet und visuell auf Normalverteilung geprüft. Das Alter der Patienten wurde als Mittelwert sowie mit 95 %-Konfidenzintervall (KI) und Standardabweichung (sd) angegeben und mangels Normalverteilung anhand eines Mann-Whitney-U-Tests getestet. Aufgrund verletzter Testannahmen wurde beim Vergleich des Alters ambulanter und stationärer Patienten auf einen χ^2^-Test mit gruppierten Alterskategorien zurückgegriffen. Kategoriale Merkmale wurden anhand von absoluten und relativen Häufigkeiten dargestellt und mittels χ^2^-Tests auf statistische Signifikanz überprüft. Ein *p*-Wert von unter 0,05 wurde als statistische Signifikanz bewertet.

## Ergebnisse

Im dem 2‑monatigen Beobachtungszeitraum wurden insgesamt 11.506 Fälle in der Notaufnahme behandelt, davon wurden 1260 Patienten (11,0 %) mit dem RTW in die Notaufnahme eingeliefert. Nur mit dem RTW wurden 76,6 % (*n* = 965) und in Notarztbegleitung 23,4 % (*n* = 295) der Patienten vorgestellt. Angaben zum Geschlecht lagen in 96,5 % der Fälle (*n* = 1216) vor. 43,4 % der Patienten (*n* = 547) waren weiblich. 44,8 % (*n* = 565) mussten stationär weiterversorgt werden, während 55,2 % (*n* = 695) ambulant verblieben. Angaben zum Alter lagen in 96,5 % der Fälle (*n* = 1216) vor. Der Altersdurchschnitt aller Patienten lag bei 54,7 Jahren (95 %-KI [53,5; 55,8], sd = 20,7), wobei ambulante Patienten mit 48,7 Jahren (95 %-KI [47,16; 50,24], sd = 20) in einem signifikanten, gerichteten Zusammenhang häufiger jünger waren als stationär aufgenommene mit 61,6 Jahren (95 %-KI [60; 63,18], sd = 19,23; *p* < 0,001).

### Prähospitale Dringlichkeitseinschätzung und Art der Weiterbehandlung

**Figure 1:**
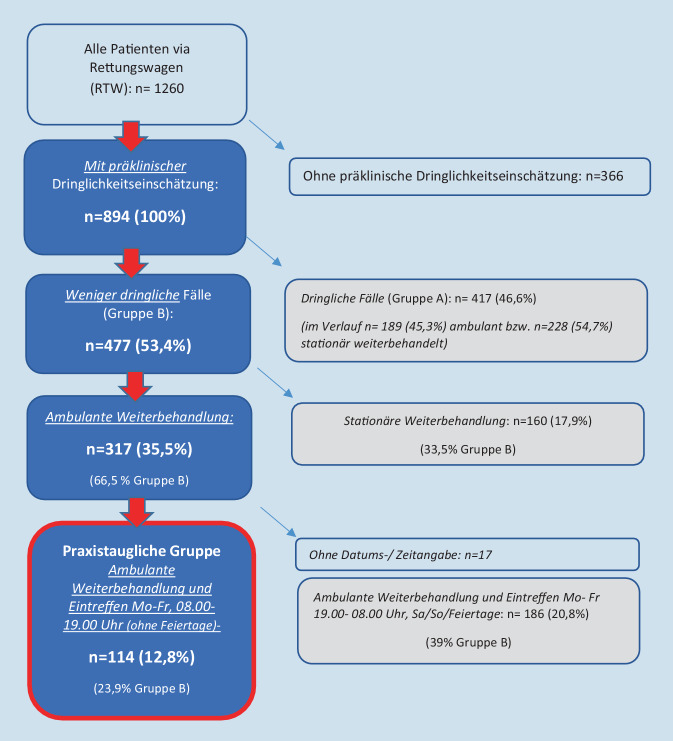

Eine prähospitale, rettungsdienstliche Dringlichkeitseinschätzung wurde bei 71,0 % (*n* = 894) der Gesamtpopulation dokumentiert (Abb. 1). Von diesen wurden 56,6 % (*n* = 506) Patienten ambulant weiterbehandelt (Tab. 1).

### „Weniger dringliche“ Fälle (Gruppe B)

53,4 % (*n* = 477) der Patienten mit dokumentierter Dringlichkeitseinschätzung entfielen in die als „weniger dringlich“ definierte Gruppe B. 30 Patienten dieser Gruppe wurde prähospital vom Notarzt gesehen (6,3 %). Die Information, ob der Notarzt auch begleitet hat oder diese nur vor Ort gesehen hat, wurde nicht ausgewertet. Das Alter der Patienten in Gruppe B war nicht signifikant unterschiedlich im Vergleich zur Gesamtpopulation (*p* = 0,124), ebenso zeigte sich kein signifikanter Unterschied in der Geschlechterverteilung zwischen den Dringlichkeitsgruppen A und B (*p* = 0,084). 66,5 % (*n* = 317) der Patienten aus Gruppe B wurden primär ambulant weiterbehandelt (Tab. 1, Abb. 1). Das entspricht 2,8 % der Gesamtzahl der im Beobachtungszeitraum in der Notaufnahme behandelten Fälle.

### „Weniger dringliche“ Fälle (Gruppe B) mit ambulanter Weiterbehandlung Montag bis Freitag, 8.00 bis 19.00 Uhr (praxistaugliche Fälle)

114 Patienten der Gruppe B wurden Montag bis Freitag (ohne Feiertage) zwischen 8.00 und 19.00 Uhr in der Notaufnahme administrativ aufgenommen und ambulant weiterbehandelt (Abb. 1). Diese Fälle könnten unter dem Aspekt der geringeren Dringlichkeit und einer ambulanten Weiterbehandlung zu den erwähnten Zeiten (aber ohne Beachtung medizinisch-inhaltlicher Aspekte) grundsätzlich in einer Arztpraxis behandelt werden (*praxistaugliche Fälle*). Das entspricht 1 % der Gesamtzahl der im Beobachtungszeitraum in der Notaufnahme behandelten Fälle (*n* = 11.506).

#### Prähospitale medizinisch-inhaltliche Einschätzung der praxistauglichen Fälle

Eine prähospitale, medizinisch-inhaltliche Bewertung konnte nach Erscheinungsbild und/oder Verletzungsregion dokumentiert werden (Tab. 2 und 3), wobei Doppelnennungen möglich waren. Insgesamt wurden *n* = 76 Erscheinungsbilder und *n* = 43 Verletzungen bei den *n* = 114 praxistauglichen Fällen dokumentiert.

**Table 2:** 

Erscheinungsbild	Anteil (Anzahl)
*Internistisch*	*60,5* *% (n* *=* *46)*
Akutes Koronarsyndrom	9,2 % (*n* = 7)
Lungenödem	0,0 % (*n* = 0)
Synkope	5,3 % (*n* = 4)
Asthma/COLD	1,3 % (*n* = 1)
BZ(Blutzucker)-Entgleisung	2,6 % (*n* = 2)
GI-Blutung	0,0 % (*n* = 0)
Sonst. int. Notfall	42,1 % (*n* = 32)
*Neurologisch*	*5,3* *% (n* *=* *4)*
TIA/Apoplex	2,6 % (*n* = 2)
Krampanfall	2,6 % (*n* = 2)
Nackensteifigkeit	0,0 % (*n* = 0)
*Sonstige*	*34,2* *% (n* *=* *26)*
Chir./orthop. Erkrankung	19,7 % (*n* = 15)
Gyn./Geburt	0,0 % (*n* = 0)
Pädiatrisch	0,0 % (*n* = 0)
Vergiftung	1,3 % (*n* = 1)
Sonstiger Notfall	13,2 % (*n* = 10)
**Gesamt**	**n** **=** **76**

**Table 3:** 

Verletzungsareal	Anteil (Anzahl)
Obere Extremitäten	32,6 % (*n* = 14)
Gesicht	20,9 % (*n* = 9)
Untere Extremitäten	16,3 % (*n* = 7)
Schädel-Hirn	9,3 % (*n* = 4)
BWS	7,0 % (*n* = 3)
HWS	4,7 % (*n* = 2)
Verbrennungen	2,3 % (*n* = 1)
Thorax	2,3 % (*n* = 1)
Becken	2,3 % (*n* = 1)
Weichteile	2,3 % (*n* = 1)
Abdomen	0,0 % (*n* = 0)
*Gesamt*	*n* *=* *43*

#### *Klinische Diagnosen* der praxistauglichen Fälle

Klinische Diagnosen waren für *n* = 112 (98 %) der *n* = 114 praxistauglichen Patienten nach Abschluss der ambulanten Behandlung in der Notaufnahme im Krankenhausinformationssystem dokumentiert (Tab. 4).

**Table 4:** 

ICD-10 (2014), Bezeichnung	Anteil (Anzahl)
I10.91 Essenzielle Hypertonie, nicht näher bezeichnet	4,5 % (*n* = 5)
M54.5 Kreuzschmerz	3,6 % (*n* = 4)
A09.9 Sonstige und nicht näher bezeichnete Gastroenteritis und Kolitis nicht näher bezeichneten Ursprungs	2,7 % (*n* = 3)
E86 Exsikkose	2,7 % (*n* = 3)
G43.1 Migräne mit Aura (klassische Migräne)	2,7 % (*n* = 3)
S61.0 Offene Wunde eines oder mehrerer Finger ohne Schädigung des Nagels	2,7 % (*n* = 3)
S93.40 Verstauchung und Zerrung des oberen Sprunggelenks	2,7 % (*n* = 3)
G40.9 Epilepsie, nicht näher bezeichnet	1,8 % (*n* = 2)
K29.6 Sonstige Gastritis	1,8 % (*n* = 2)
M54.2 Zervikalneuralgie	1,8 % (*n* = 2)
M54.4 Lumboischialgie	1,8 % (*n* = 2)
R00.2 Palpitationen	1,8 % (*n* = 2)
R42 Schwindel und Taumel	1,8 % (*n* = 2)
R51 Kopfschmerz	1,8 % (*n* = 2)
R55 Synkope vasovagal	1,8 % (*n* = 2)
S00.85 Oberflächliche Verletzung sonstiger Teile des Kopfs, Prellung	1,8 % (*n* = 2)
S06.0 Commotio cerebri	1,8 % (*n* = 2)
S13.4 Verstauchung und Zerrung der Halswirbelsäule	1,8 % (*n* = 2)
*Diagnosen mit weniger als n* *=* *2*	*58,9* *% (n* *=* *66)*
*Gesamt*	*n* *=* *112*

### Vergleich der prähospitalen mit der klinischen Dringlichkeitseinschätzung

**Figure 2:**
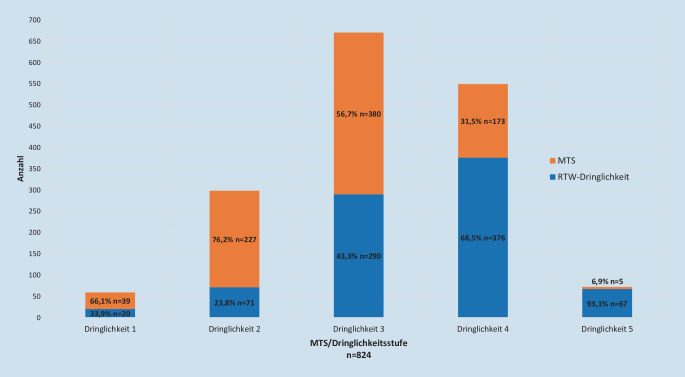

Bei 65,4 % (*n* = 824) der Fälle war sowohl eine prähospitale als auch eine klinische Dringlichkeitseinschätzung dokumentiert (Abb. 2). In 34,6 % (*n* = 285) der Fälle zeigte sich eine Übereinstimmung in den jeweils 5‑stufigen Dringlichkeitseinschätzungsskalen. Bei 9,5 % (*n* = 78) der Patienten war die prähospitale Einschätzung dringlicher als die Einschätzung im MTS. Bei 55,9 % (*n* = 461) der Patienten fand in der MTS eine dringlichere Eingruppierung statt.

**Figure 3:**
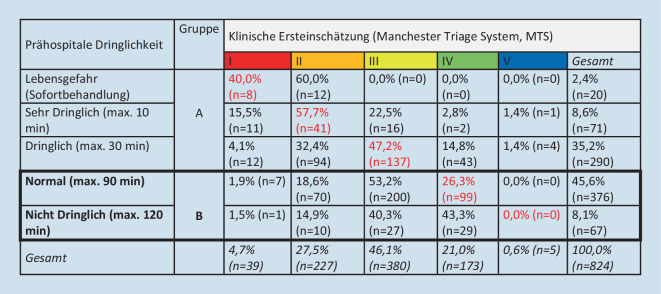

Von *n* = 443 der „weniger dringlichen“ Fälle (Gruppe B), bei denen auch eine innerklinische Ersteinschätzung dokumentiert war, wurden 71,1 % (*n* = 315) in der Notaufnahme/im MTS „hochgestuft“: 72,1 % (*n* = 227) in Stufe 3 (gelb); 25,4 % (*n* = 80) in Stufe 2 (orange) und 2,5 % (*n* = 8) in Stufe 1 (rot; Abb. 3).

Von den *n* = 114 Patienten der praxistauglichen Subgruppe der „weniger dringlichen“ Fälle war bei *n* = 110 Fällen eine MTS-Einschätzung dokumentiert. Insgesamt wurden *n* = 70 Fälle (63,6 %) hochgestuft, wobei *n* = 55 (50,0 %) in die MTS-Stufe III und *n* = 15 (13,6 %) in die MTS-Stufe II eingeschätzt wurden. Von den der „dringlichen“ Gruppe zugeordneten Fälle wiederum wurden *n* = 14 in der potenziellen Praxisöffnungszeit in der Notaufnahme ambulant behandelt und in der Manchester Triage in der Dringlichkeit herabgestuft (*n* = 12 in MTS IV und *n* = 2 in MTS V).

## Diskussion

Der Sachverständigenrat zur Begutachtung der Entwicklung im Gesundheitswesen empfiehlt in seinem Gutachten zur bedarfsgerechten Steuerung der Gesundheitsversorgung im Jahr 2018 (Empfehlung 1015, [4]), dass der Rettungsdienst am Einsatzort auch entscheiden können solle, geeignete Patienten zur Behandlung direkt zu niedergelassenen Ärzten zu transportieren. In der hier vorliegenden, retrospektiven Untersuchung von prähospitalen und klinischen Routinedaten von Patienten, die mit dem Rettungstransportwagen in die Notaufnahme eines großstädtischen, universitären Maximalversorgers eingeliefert wurden, sollte primär analysiert werden, welcher Anteil am Gesamtaufkommen dieser Patienten sicher und sinnvoll direkt in die vertragsärztliche Versorgung hätte disponiert werden können. Als Surrogatparameter für eine sichere Zuweisung wurde die in der Präklinik eingeschätzte Dringlichkeit bestimmt, für eine sinnvolle Zuweisung die ambulante Weiterbehandlung.

### Dringlichkeit

**Figure 4:**
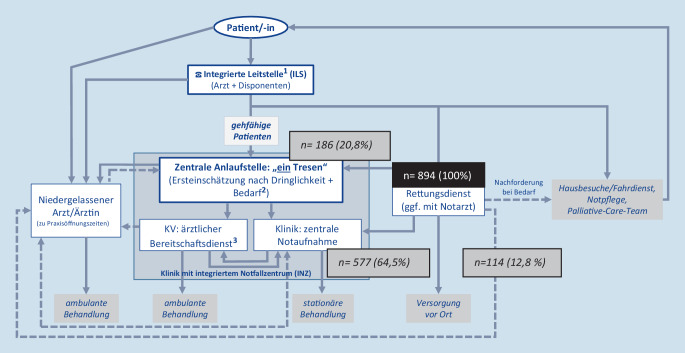

Im Untersuchungszeitraum konnte im Rettungsdienstprotokoll der RTW der Berliner Feuerwehr eine 5‑stufige Dringlichkeitseinschätzung erfolgen, die in den zeitlichen Einschätzungskategorien den Vorgaben des in der Notaufnahme verwendeten Manchester Triage System entsprach, ohne jedoch entsprechende Parameter oder Diagramme für die Zuordnung in der Dokumentation vorzugeben. Sie war mit der Intention entstanden, der Notaufnahme bereits eine prähospitale Dringlichkeitseinschätzung der Behandlung kommunizieren zu können bzw. Patienten entsprechend voranzumelden. Eine Studie aus den Jahren 2010/2011, in deren Rahmen geschulte Rettungsdienstmitarbeiter einer Berliner Feuerwache eine Dringlichkeitseinschätzung vornahmen, zeigte eine gute Übereinstimmung mit der nachgelagerten Ersteinschätzung einer universitären Notaufnahme [12]. Die Berliner Feuerwehr weitete die Schulungen jedoch nicht aus. Auf Basis dieser prähospitalen Dringlichkeitseinschätzung wurde, ungeachtet der Art der Erkrankung oder Verletzung, die Gesamtkohorte durch die Autoren in „dringliche“ und „weniger dringliche Fälle“ geteilt, wobei Fälle, deren Dringlichkeit minimal 30 min bis max. 120 min bis zu einem Arztkontakt vorsah, als potenziell zum primären Transport in eine Arztpraxis geeignet angesehen wurden. Würde man nur dieser Dringlichkeitsgruppierung folgen, könnten über 50 % der mit dem RTW im Untersuchungszeitraum der Notaufnahme zugewiesenen Fälle primär als weniger dringliche Fälle potenziell in eine Niederlassung disponiert werden, wenn diese 24/7 verfügbar wären bzw. wenn außerhalb der Praxisöffnungszeiten die Patienten primär einem „gemeinsamen Tresen“ zugeführt würden (Abb. 4).

Bei über 70 % der auf Basis der prähospitalen Einschätzung als „weniger dringlich“ kategorisierten Patienten erfolgte in der Klinik eine Heraufstufung der Dringlichkeit im Manchester Triage System, was im Fall einer primären Zuweisung durch den nichtnotärztlichen Rettungsdienst in eine Praxis eine Patientengefährdung darstellen könnte.

So stellten Sefrin et al. bei der Auswertung rein prähospitaler Daten von 3127 bundesweiten Einsätzen des Rettungsdiensts des Deutschen Roten Kreuzes fest, dass es sich bei nur rund einem Drittel der aufgrund einer Notfallmeldung disponierten Rettungsfahrten nicht um Notfallpatienten im eigentlichen Sinne handelt [10], deren Versorgung von den Autoren im vertragsärztlichen Bereich gesehen wurde.

### Weiterbehandlung

Zwei Drittel der „weniger dringlichen“ Fälle konnten ambulant weiterbehandelt werden und hätten somit theoretisch sinnvoll primär in eine fachärztliche Niederlassung transportiert werden können. Jeder Vierte dieser Patienten wurde Montag bis Freitag, jeweils zwischen 9.00 bis 18.00 Uhr in der Notaufnahme ambulant behandelt, also in einem Zeitraum, in dem der Rettungsdienst insbesondere im großstädtischen Bereich auf zahlreiche und inhaltlich diverse Facharztniederlassungen zurückgreifen könnte. Aus einer Dringlichkeitseinschätzung lassen sich allerdings keine sicheren Vorabaussagen zur Art der Weiterbehandlung ableiten [8, 11], selbst nicht aus einer notärztlichen [1]. Von den „weniger dringlichen“ Patienten wurde in unserer Studie jeder Dritte stationär aufgenommen. Die primäre Zuweisung „weniger dringlicher“ Fälle durch den nichtnotärztlichen Rettungsdienst in eine Praxis erscheint daher auch unter dem Aspekt der Sinnhaftigkeit schwierig und sekundäre private oder auch rettungsdienstliche Transporte bei stationärer Behandlungsbedürftigkeit müssten eingeplant werden.

### Ressourcen

Die in der Notaufnahme zur Diagnostik, Behandlung und somit auch zur Festlegung der notwendigen Weiterbehandlung der „wenig dringlichen“ Patienten eingesetzten Ressourcen (technisch apparativ, aber auch Verweildauer) wurden in unserer Studie nicht ausgewertet. Es bleibt daher offen, ob diese überhaupt regelhaft auch in der Praxis bereitgestellt werden können. Da in der präklinischen Notfallmedizin der Auswahl der bestmöglichen Anschlussversorgung gemäß dem Stand der medizinischen Wissenschaft auch in haftungsrechtlicher Hinsichtlich eine stark gewachsene Bedeutung zukommt, erscheint der sichere Ex-ante-Ausschluss einer umfassenderen Behandlungsbedürftigkeit durch den Rettungsdienst weder zuverlässig noch umfassend möglich. Betrachtet man medizinisch-inhaltliche Aspekte, lässt sich daher mutmaßen, dass der Rettungsdienst bei prähospital dokumentierten Erscheinungsbildern wie „ACS“ oder „TIA/Apoplex“ (Tab. 2) trotz gering eingeschätzter Dringlichkeit einen Transport in die Notaufnahme durchführen würde, auch wenn sich diese in der Konstellation selten retrospektiv als Diagnose bestätigen (Tab. 4). Gleiches ist beim Verletzungsmuster „Schädel-Hirn“ anzunehmen (Tab. 3), da vom Rettungsdienst bereits die eventuelle Notwendigkeit einer CT-Untersuchung antizipiert werden dürfte. Auch die in der Klinik für diese Patientenkohorte dokumentierten Hauptdiagnosen (Tab. 4) lassen in der deutlichen Mehrheit der Fälle erkennen, dass diese erst nach umfassender Diagnostik und dem Ausschluss gravierenderer Befunde sicher erhoben werden konnten.

Es ist daher davon auszugehen, dass der Anteil der Patienten, die auch sinnvoll primär in der Praxis behandelt werden könnten, noch geringer einzuschätzen ist als dargestellt (Abb. 4).

Grundsätzlich ist außerdem festzuhalten, dass diese relativ hohen Raten an durch den Rettungsdienst disponierten Patienten, die potenziell unter Umgehung der Notaufnahme zu versorgen wären, nur zu einer unbedeutenden Entlastung derselben führen würden. So macht die für die Studie definierte gesamte Kategorie der „weniger dringlichen“ Fälle des Rettungsdiensts mit ambulanter Weiterbehandlung weniger als 3 % der Gesamtzahl der im 2‑monatigen Beobachtungszeitraum in der großstädtischen, universitären Notaufnahme behandelten Fälle aus. Schränkt man weiter auf die angenommenen Praxisöffnungszeiten ein, beträgt der Anteil in unserer Studie nur noch 1 %, wobei dieser sicherlich abhängig von lokalen bzw. regionalen infrastrukturellen Gegebenheiten (Stadt vs. Land, Versorgungsstufe der Notaufnahme, Dichte der niedergelassenen Fachärzte) schwanken kann.

Als sekundäres Ergebnis unserer Untersuchung zeigte sich, dass die prähospitale Dringlichkeitseinschätzung als Basis der sicheren Zuweisung in die Praxis in unserer Studie nur bei einem Drittel der Fälle mit der Ersteinschätzung in der Notaufnahme, die regelhaft durch geschultes Personal erfolgte, übereinstimmte. Über die Hälfte der prähospital als „normal“ oder „nicht dringlich“ kategorisierten Fälle wurde in der Notaufnahme höher eingestuft, wobei der größte Anteil dieser Patienten (70 %) als „gelb“ (= Arztkontakt sollte in den folgenden 30 min erfolgen, MTS III) im Manchester Triage System eingeschätzt wurde, was prinzipiell natürlich auch in der Niederlassung zu erfüllen wäre, insbesondere dann, wenn sich Praxen an speziellen Tagen auf diese Aufgabe vorbereiteten. In der praxistauglichen Gruppe wurden über 60 % der Fälle in der Klinik dringlicher eingeschätzt, die Hälfte in MTS III, aber auch über 10 % in MTS-Stufe II, was einen Arztkontakt innerhalb von 10 min erfordern würde und bei Transport in die Niederlassung eine Gefährdung der Patientensicherheit darstellen könnte. Die Angemessenheit der jeweiligen Dringlichkeitseinschätzung wurde in der Studie nicht objektiviert. Neben der zeitlichen Dynamik könnte die Diskrepanz zwischen prähospitaler und klinischer Einschätzung auch darin begründet sein, dass das Rettungsdienstpersonal im Gegensatz zum Notaufnahmepersonal nicht speziell in der 5‑stufigen Dringlichkeitseinschätzung des Patienten am Einsatzort geschult worden war und Kriterien zur Einschätzung nicht in der Rettungsdienstdokumentation hinterlegt waren. Iversen et al. konnten allerdings bereits zeigen, dass die Verwendung einer „Blickeinschätzung“ in Kombination mit der Befragung zur Hauptbeschwerde einer 5‑stufigen Einschätzungsskala (Danish Emergency Process Triage, DEPT) zumindest in Bezug auf die Vorhersage der 48 h-Mortalität überlegen sein kann und von dessen Einstufungen deutlich abweicht [6]. Zur Zuweisung von Patienten in alternative Versorgungsangebote müssen zunächst Handlungsleitfäden für die Entscheidungsfindung des rettungsdienstlichen Fachpersonals entwickelt und validiert werden. Für den Rettungsdienst im Land Berlin wurden ihm Rahmen der COVID-19-Pandemie erste Ansätze hierzu untersucht [2]. Neben der notwendigen medizinischen und medikolegalen Absicherung entsprechender Zuweisungsentscheidungen stellen auch die vielfältigen und wechselnden Behandlungsressourcen in den Arztpraxen eine erhebliche organisatorische Herausforderung dar, wobei initiale Zuweisungsentscheidungen nicht erst vor Ort getroffen werden. Die Notrufabfrage in der Leitstelle der Berliner Feuerwehr erfolgt unter Anwendung des Priority Dispatch Systems, das die weltweit umfangreichsten Möglichkeiten zur standardisierten Notrufabfrage dringlicher und nicht dringlicher medizinischer Hilfeersuchen bietet [7], wobei nicht dringliche Fälle an den kassenärztlichen Bereitschaftsdienst weitergleitet werden können. Durch die Kassenärztliche Vereinigung wird als telefonisches Einschätzungsinstrument für die Anrufe auf der Nummer „116117“ die Software SmED (Strukturiertes medizinisches Ersteinschätzungsverfahren für Deutschland, Kassenärztliche Bundesvereinigung, Berlin) verwendet. Mit SmED soll die Dringlichkeit (Zeitpunkt der Versorgung: *Notfall, schnellstmögliche medizinische Versorgung, medizinische Versorgung heute, medizinische Behandlung eilt nicht*) und die richtige Versorgungsebene (*Rettungsdienst, Notaufnahme, Arzt/ärztlicher Bereitschaftsdienst oder ärztliche Telefonkonsultation*) zur weiteren Abklärung der durch den Patienten geäußerten Beschwerden eingeschätzt werden können [5]. In einem Projekt (SaN) der Kassenärztlichen Vereinigung in Hessen erfolgt die prähospitale Erprobung von SmED als Einschätzungsinstrument in Kombination mit IVENA (Interdisziplinärer Versorgungsnachweis, IVENA eHealth, mainis IT-Service GmbH, Offenbach), in dem Partnerpraxen ihre Aufnahmebereitschaft in Echtzeit abbilden können (https://kv-innovationsscout.de/projekt/san-projekt, zuletzt besucht am 16.05.2021; [9]). Es erscheint hier zunächst plausibel, dass mit der Abfrage in der Leitstelle bereits weitgehend sichergestellt werden sollte, dass der überlastete Rettungsdienst nur zu stationären Notfällen disponiert wird. Allerdings wurde im Kontext von COVID-19 auch hier ein Digitalisierungsfortschritt erreicht und ein Zusatzmodul von IVENA (siehe oben) erfolgreich erprobt, das wiederum nur die Disposition in die richtige Klinik, aber nicht in eine Arztpraxis zum Inhalt hat [3]. Darüber hinaus ist wenig bekannt, welche Kompetenzen im Rettungsdienst erforderlich wären, um vor Ort gegebenenfalls auch über den Verbleib in der Häuslichkeit zu entscheiden. Zu dieser Frage führen wir zurzeit in Kooperation mit der Berliner Feuerwehr eine öffentlich geförderte Studie durch (EMAPREPARE; https://emanet.charite.de/, zuletzt besucht am 16.05.2021). Im Hinblick auf die Integration von KV-Strukturen am Krankenhaus im Sinne von INZ erscheint es uns für rettungsdienstliche Fälle somit sinnvoller, diese zunächst primär in der Notaufnahme zu versorgen und ggf. sekundär in die Notdienstpraxis des jeweiligen Standorts weiterzuleiten.

## Limitationen

Limitierend in unserer Studie ist die monozentrische Betrachtung über einen Zeitraum von nur 2 Monaten. Unter der Annahme, dass der Rettungsdienst vorwiegend schwerere Fälle in eine Universitätsklinik einliefert, könnte gemutmaßt werden, dass in anderen Krankenhäusern die Quote der potenziell primär in eine Praxis zu disponierenden Fälle höher liegt. Die Fallzahlen im auf die Monate bezogenen Betrachtungszeitraum der Studie (01.05. bis 30.06.2014, *n* = 11.506) stiegen auf ein Maximum von *n* = 12.293 im Jahr 2016 und lagen 2019 mit *n* = 11.866 somit um 360 Fälle höher als im Betrachtungsjahr. Aktuell ist in den Protokollen der Berliner Feuerwehr keine am MTS orientierte, fünfstufige Dringlichkeitseinschätzung mehr vorgesehen, sodass keine vergleichbaren, prähospitalen Daten für den Abgleich mit der Dringlichkeitseinschätzung in der Notaufnahme herangezogen werden können. Es erfolgte keine retrospektive Einschätzung, ob ggf. auch eine Versorgung vor Ort durch den Rettungsdienst ausreichend gewesen wäre.

## Fazit für die Praxis

- Der Rettungsdienst könnte eine großstädtische universitäre Notaufnahme durch die primäre Disposition geeigneter Patienten in die Facharztniederlassung um ca. 1 % ihrer Gesamtbehandlungszahl entlasten.
- Medizinisch-inhaltliche Aspekte und in der Praxis vorzuhaltende Ressourcen dürften die Zahl geeigneter Patienten weiter einschränken.
- Es muss mit einer prähospitalen Unterschätzung der Dringlichkeit gerechnet werden.
- Zusammenfassend erscheint die Etablierung eines solchen Prozesses unter dem zentralen Aspekt einer Entlastung der Notaufnahmen unbedeutend und wirkungslos, mit einem hohen logistischen Aufwand verbunden und potenziell patientengefährdend zu sein.
