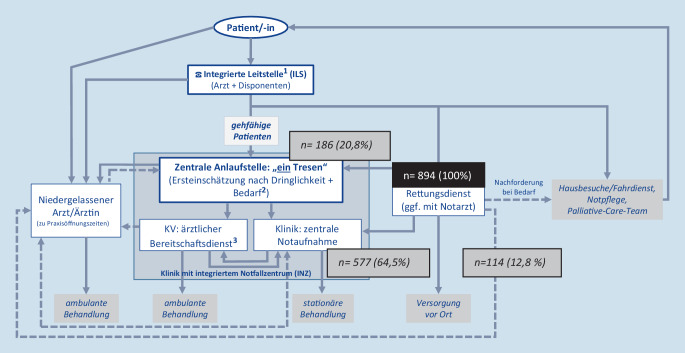# Erratum zu: Mit dem Rettungsdienst direkt in die Arztpraxis – eine wirkungsvolle Entlastung der Notaufnahmen?

**DOI:** 10.1007/s00063-021-00874-5

**Published:** 2021-10-04

**Authors:** Tobias Lindner, Alessandro Campione, Martin Möckel, Cornelia Henschke, Janosch Dahmen, Anna Slagman

**Affiliations:** 1 Notfall- und Akutmedizin, Charité – Universitätsmedizin Berlin Campus Virchow-Klinikum und Campus Mitte, Augustenburger Platz 1, 13353 Berlin, Deutschland; 2 Fachgebiet Management im Gesundheitswesen, Technische Universität Berlin, Berlin, Deutschland; 3 Fakultät für Gesundheit, Department Humanmedizin, Universität Witten/Herdecke, Witten, Deutschland; 4 Ärztliche Leitung Rettungsdienst, Berliner Feuerwehr, Berlin, Deutschland

**Erratum zu:**

**Med Klin Intensivmed Notfmed 2021**

10.1007/s00063-021-00860-x

In diesem Artikel wurde die Abb. 4 falsch wiedergegeben. Die Abbildung hätte wie hier dargestellt erscheinen müssen. Der Originalbeitrag wurde korrigiert.

**Figure 1:**